# Use of a miniature diamond-anvil cell in high-pressure single-crystal neutron Laue diffraction

**DOI:** 10.1107/S2052252516000725

**Published:** 2016-02-26

**Authors:** Jack Binns, Konstantin V. Kamenev, Garry J. McIntyre, Stephen A. Moggach, Simon Parsons

**Affiliations:** a Australian Nuclear Science and Technology Organisation, New Illawarra Road, Lucas Heights, NSW 2234, Australia; b EaStCHEM School of Chemistry and Centre for Science at Extreme Conditions, The University of Edinburgh, The King’s Buildings, West Mains Road, Edinburgh EH9 3FJ, United Kingdom; c School of Engineering and Centre for Science at Extreme Conditions, The University of Edinburgh, Erskine Williamson Building, The King’s Buildings, Peter Guthrie Tait Road, Edinburgh EH9 3JJ, United Kingdom

**Keywords:** high pressure, neutron diffraction, Laue diffraction

## Abstract

The first high-pressure neutron diffraction study in a miniature diamond-anvil cell of a single crystal of size typical for X-ray diffraction is reported. This is made possible by modern Laue diffraction using a large solid-angle image-plate detector. An unexpected finding is that even reflections whose diffracted beams pass through the cell body are reliably observed, albeit with some attenuation. The cell body does limit the range of usable incident angles, but the crystallographic completeness for a high-symmetry unit cell is only slightly less than for a data collection without the cell. Data collections for two sizes of hexamine single crystals, with and without the pressure cell, and at 300 and 150 K, show that sample size and temperature are the most important factors that influence data quality. Despite the smaller crystal size and dominant parasitic scattering from the diamond-anvil cell, the data collected allow a full anisotropic refinement of hexamine with bond lengths and angles that agree with literature data within experimental error. This technique is shown to be suitable for low-symmetry crystals, and in these cases the transmission of diffracted beams through the cell body results in much higher completeness values than are possible with X-rays. The way is now open for joint X-ray and neutron studies on the same sample under identical conditions.

## Abstract 2

High-pressure single-crystal neutron Laue diffraction yields data suitable for fully anisotropic structure refinement, allowing joint X-ray and neutron studies of exactly the same sample. Remarkably, data completeness is similar to ambient-pressure measurements, despite the presence of a pressure cell.

## 1. Introduction   

Diffraction methods can provide the highest-quality structural information about a crystal on the atomic scale and much work has been carried out to adapt X-ray and neutron diffraction techniques to a variety of challenging sample environments, including high pressure (McMahon *et al.*, 2013 ▸; Guthrie, 2015 ▸). X-ray diffraction benefits from the strong photon–electron interaction as well as excellent and relatively inexpensive laboratory sources which can be complemented by very-high-intensity synchrotron sources. In particular, improvements in synchrotron technology have led to the development of dedicated high-pressure beamlines (McMahon, 2015 ▸). In many ways neutron diffraction is the reverse; all sources are located at central facilities and are weak compared even to laboratory X-ray tubes. However, neutrons can have several advantages over X-rays, the most relevant to high-pressure crystallography being their greater sensitivity to low-*Z* atoms, particularly hydrogen, and their greater penetrability through extreme sample environments.

High-pressure neutron powder diffraction has been successfully applied to the discovery of new phases of simple hydrates (Loveday *et al.*, 2001 ▸; Fortes *et al.*, 2007 ▸) and ices (Nelmes *et al.*, 2006 ▸; Fortes *et al.*, 2012 ▸). Amongst larger molecular systems it has been applied to the study of polymorphism in amino acids (Moggach *et al.*, 2006 ▸; Funnell *et al.*, 2010 ▸) and explosives (Davidson *et al.*, 2008 ▸).

The Paris–Edinburgh (PE) cell developed in the 1990s has become a standard workhorse of high-pressure neutron powder diffraction and is capable of experiments up to 10 GPa with sample volumes of approximately 100 mm^3^ (Besson *et al.*, 1995 ▸). Replacing the standard tungsten carbide anvils with sintered diamond allows the maximum pressure to be increased to 25 GPa, although using a smaller sample volume of approximately 35 mm^3^ (Besson *et al.*, 1995 ▸; Klotz *et al.*, 1995 ▸). These studies have also exploited the construction of new beamlines and instruments dedicated to high-pressure neutron diffraction (Boehler *et al.*, 2013 ▸; Guthrie *et al.*, 2013 ▸; ISIS, 1996 ▸, 1997 ▸). However, high-pressure neutron powder diffraction methods have two significant and well known disadvantages with respect to single-crystal techniques: the loss of information due to peak overlap which is exacerbated by strain broadening, and the requirement to deuterate the sample. Deuteration can present a significant synthetic challenge for molecules of even modest complexity and can occasionally induce structural changes in molecular crystals by altering the vibrational properties of individual molecules and of the crystal as a whole. As a result the thermodynamic and mechanical properties of deuterated and undeuterated crystals can be significantly different (Crawford *et al.*, 2009 ▸). For high-pressure neutron diffraction studies of hydrogenated samples, single crystals are essential.

High-pressure single-crystal neutron diffraction experiments have been conducted with both time-of-flight (Bull *et al.*, 2009 ▸) and monochromatic sources (Bull *et al.*, 2011 ▸) up to 10 GPa using the VX Paris–Edinburgh press (Klotz *et al.*, 2004 ▸). Time-of-flight data were collected on the Laue diffractometer SXD at the ISIS pulsed neutron source on KD_2_PO_4_ and squaric acid (D_2_C_4_O_4_) at 5 and 7.5 GPa (Bull *et al.*, 2009 ▸). Further high-pressure data using a monochromatic neutron beam were collected on squaric acid on the D9 single-crystal diffractometer at the Institut Laue–Langevin (ILL) at 3.5 and 10 GPa. Measurements were carried out sequentially stepping through the reflections with collection times typically 15–20 min per reflection, increasing to up to 1 h for high-resolution reflections (Bull *et al.*, 2011 ▸). The geometry of the PE-cell constrains the application of this technique to situations where the quasi-two-dimensional access is sufficient for structural refinements. Diamond/moissanite-anvil cells with much greater angular access have also been utilized in studies using both time-of-flight (Bull *et al.*, 2009 ▸) and steady-state Laue methods (McIntyre *et al.*, 2005 ▸). Successful as these neutron experiments have been, complementing high-pressure X-ray data with high-pressure neutron data is still fraught with the technical challenge to obtain identical conditions.

The adaption of neutron-sensitive image plates with high spatial resolution has sparked a renaissance in the application of neutron Laue methods at reactor sources with LADI and VIVALDI, both at the ILL (Cipriani *et al.*, 1996 ▸; McIntyre *et al.*, 2006 ▸), and now with KOALA at ANSTO (Edwards, 2011 ▸), which exploit the excellent match between attainable resolution and the low divergence of the guides on which the Laue diffractometers are located. These characteristics allow the study of single crystals with volumes several orders of magnitude smaller than usually required for neutron diffraction, and the principal aim of this paper is to demonstrate that this sensitivity enables single-crystal neutron diffraction data to be collected using samples contained in diamond-anvil cells of the type also used for X-ray measurements, opening the way for joint spectroscopic and diffraction studies using neutrons and X-rays on exactly the same sample.

By using the full polychromatic beam, the Laue technique mitigates some of the difficulties in collecting data from small samples at reasonable rates. The cylindrical image-plate detectors offer distinct advantages over electronic detectors particularly for high-pressure collections. The small point-spread function leads to Bragg spots which are approximately equal to the projected form of the crystal, regardless of intensity. Given the disparity in crystal volume between the anvils and the sample, this usually facilitates separation of the corresponding three sets of Bragg reflections. A further advantage of the image-plate detectors is the ability to overexpose strong reflections without detrimental effects on the detector. Therefore, very strong and very weak scattering can be recorded at the same time and in very close proximity (McIntyre *et al.*, 2005 ▸).

Hexamethylenetetramine (or hexamine, HMT, C_6_H_12_N_4_) was chosen as the sample material for the initial experiments. The crystal structure was first determined by Dickenson & Raymond (1923 ▸) and has since been the model crystalline system for a variety of fundamental diffraction studies including charge density and neutron diffraction (Duckworth *et al.*, 1970 ▸; Terpstra *et al.*, 1993 ▸). HMT crystallizes in space group 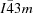, with *a* = 7.028 (2) Å (300 K), occupying a site with 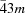 symmetry. The asymmetric unit thus consists of only three atoms, all of which lie on symmetry-constrained positions giving a total of 14 parameters for a fully anisotropic refinement.

Further experiments were carried out using the naturally occurring amino acid l-arginine (referred to as **R**, C_6_H_14_N_4_O_2_·2H_2_O). l-Arginine crystallizes from aqueous solution as a dihydrate in an orthorhombic unit cell, space-group symmetry *P*2_1_2_1_2_1_, *a* = 5.6243 (1), *b* = 11.8081 (3) and *c* = 15.5406 (3) Å, *V* = 1032.09 (4) Å^3^. The crystal structure was first reported by Karle & Karle (1964 ▸), with a subsequent neutron diffraction study carried out by Lehmann *et al.* (1973 ▸) as part of the Brookhaven series of amino-acid structure determinations. The side-chain of l-arginine consists of three aliphatic carbon atoms terminated by a guanidinium group. In the dihydrate the two water molecules link the repeat units in an infinite ordered chain. This more complex, lower-symmetry structure requires a total of 289 parameters for a fully anisotropic refinement.

## 2. Methods   

### 2.1. Miniature diamond-anvil cell   

**Figure 1:**
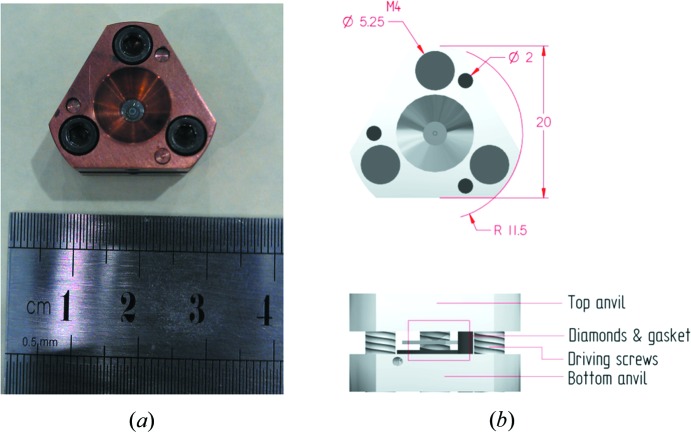
(*a*) Photograph of the miniature DAC; (*b*) diagram of the miniature DAC including cell dimensions in mm.

The miniature diamond-anvil cell (mini-DAC, Fig. 1 ▸) was a Merrill–Bassett type (Merrill & Bassett, 1974 ▸) using beryllium–copper alloy for the construction of the cell body and the backing plate. The alloy used was BERYLCO-25, chosen for its low thermal contraction and high thermal conductivity, with composition 1.8–2.0 wt% Be, a maximum 0.6 wt% Ni, Co and Fe, and the remainder Cu. The design was optimized by using finite-element analysis in order to evaluate the strength of the cell and its individual components. The diamond anvils were Boehler–Almax cut, 3 mm high with 1 mm [001] culet faces set into the body by 1.5 mm (Moggach *et al.*, 2008 ▸). The thickness of each half of the cell body was 5 mm, with a height of 20 mm. Alignment was established with three 2 mm diameter Be–Cu guide pins. The backing plate gave optical access through the rear of the anvils with an opening half angle, ω, of 39°.

The small size of the cell allows it to be mounted within the cryostat shields on the KOALA Laue single-crystal diffractometer on the OPAL reactor at ANSTO.

Beyond its reduced size and use of BERYLCO-25, this cell does not differ in construction from the design described by Merrill & Bassett (1974 ▸) or Moggach *et al.* (2008 ▸). The cell can also be mounted on a standard goniometer head for X-ray diffraction measurements. The optical access afforded by the large opening angle and transparent diamonds allows measurement of pressure by ruby fluorescence as well as other *in situ* spectroscopic measurements (Piermarini *et al.*, 1975 ▸).

### 2.2. Crystallization and data collection geometry   

Crystals of undeuterated HMT-h_12_ were selected from commercial samples supplied by Sigma Aldrich. Crystals of undeuterated l-arginine dihydrate were grown by slow evaporation of a saturated aqueous solution of l-arginine following literature growth studies (Mallik & Kar, 2005 ▸).

**Figure 2:**
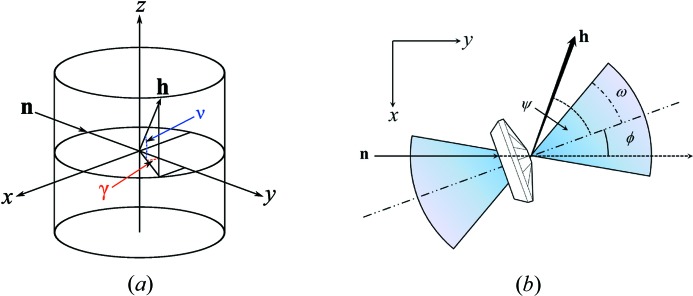
(*a*) Schematic of the KOALA instrument. The incident beam **h** travels along the instrument *y* axis. During an experiment the crystal is rotated about the vertical *z* axis by an angle Δϕ between successive patterns. Each reflection **h** is characterized by the horizontal and vertical polar angles γ, ν. (*b*) The angle ϕ orients the mini-DAC with respect to the incident beam, ψ is the angle a reflection **h** makes with the mini-DAC axis. The geometry of the mini-DAC creates an opening half angle ω which limits the direction of the incident beam.

The KOALA diffractometer consists of a cylindrical detector faced by neutron-sensitive image plates located at the end of a thermal neutron guide; it is essentially a copy of VIVALDI (McIntyre *et al.*, 2006 ▸). The incident unmonochromated thermal-neutron beam has a Maxwellian distribution of wavelengths between 0.5 Å and 4 Å (3.27–5.11 meV). The detector area subtends ±144° in the horizontal plane and ±52° out of the plane at the sample, and in a typical experiment four to ten diffraction patterns are collected at different angles of rotation (ϕ) about the instrument’s vertical *z*-axis. In the instrument coordinate system, shown in Fig. 2 ▸(*a*), the origin lies at the sample, the *z* axis is vertical pointing upwards along the cylindrical axis of the detector, the incident beam travels along the positive *y* axis with the *x* axis making a right-handed set. With the mini-DAC mounted it is convenient to define the rotation angle ϕ = 0° where the cell axis lies along the incident beam, **n**.

The geometry of the cell defines an opening half angle ω, and data are collected with |ϕ| < ω to maximize incident flux at the sample and avoid high background due to scattering if the incident beam passes through the cell body. The incident beam was collimated to a diameter of 1 mm at a distance of 22 cm before the sample.

The angle which the scattered ray, **h**, makes with the cell axis is denoted ψ. If ψ ≤ ω, the diffracted beam passes only through a diamond, but if ψ > ω, the diffracted beam passes through a diamond and the cell body. The direction of a diffracted beam is defined with respect to the instrument coordinate system by two cylindrical polar angles: γ in the horizontal and ν in the vertical planes, with both equal to zero along the incident beam. A general schematic of the KOALA instrument is given in Fig. 2 ▸(*a*), and the reference angles about the mini-DAC are shown in Fig. 2 ▸(*b*).

### 2.3. Sample centring   

**Figure 3:**
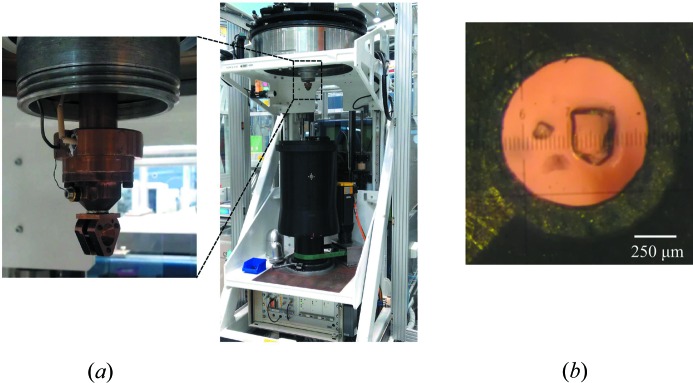
(*a*) KOALA quasi-Laue diffractometer on the OPAL reactor ANSTO, inset shows the mini-DAC mounted on the instrument; (*b*) microscope photograph of the crystal B of HMT along with a chip of ruby in the DAC gasket.

To centre a sample mounted in the mini-DAC, the cell was rotated around the vertical cylindrical axis to view between the two diamonds and the approximate sample height and off-set determined optically. A series of 18 exposures each of 1 h with *x*, *y* and *z* off-sets displaced by ± 0.5 and ± 1.0 mm from their initial values were then collected. The intensities of several intense sample spots were monitored at each position and the off-sets where these were maximized taken as the centre. The sample position along *z* was re-optimized before the collections at 150 K to account for thermal contraction of the sample holder. Fig. 3 ▸(*a*) shows the mini-DAC mounted on the KOALA instrument.

### 2.4. Data collection   

Ambient-pressure experiments without the mini-DAC were carried out to gauge the effects of sample size. Data were collected at 300 K using a crystal of HMT-h_12_ of dimensions 0.4 × 0.4 × 0.3 mm (crystal **A**), and a smaller sample measuring 0.3 × 0.2 × 0.15 mm (crystal **B**), which was small enough that it could be accommodated within the gasket of a mini-DAC (Fig. 3 ▸ *b*). These data sets will be designated **A**_300_ and **B**_300_, respectively. Both **A**_300_ and **B**_300_ consisted of four patterns collected for approximately 4 h each. Crystal **B** was also cooled to 150 K and four patterns were recorded each for 12 h (data-set **B**_150_). Rotation steps of 20° were used for all three collections.

Crystal **B** was then loaded into the mini-DAC using a circular steel gasket of thickness 250 µm, radius 5 mm, and an internal diameter 800 µm, with Fluorinert FC75 as a hydrostatic medium. Pseudo-Kossel lines were observed in the images as a result of increased mosaic spread in the near-perfect anvils while under stress (Binns *et al.*, 2016 ▸). By reducing primary extinction, the scattering power of the diamonds increases and the strongest diamond reflections in effect become secondary sources. The presence of pseudo-Kossel lines indicated that the sample pressure was ∼ 0.25 GPa (Loveday *et al.*, 1990 ▸). The applied pressure was low in order to validate the structural parameters against ambient pressure data, separating the effects of placing the sample within the cell from the effects of application of pressure.

The high-pressure, room-temperature data set on crystal **B**, **B**_DAC,300_, consisted of six patterns collected at ϕ = −30°, −20°, −10°, 0°, 10° and 20°. Exposure times were 12 h for the first five and 8 h for the final pattern. Low-temperature, high-pressure data, **B**_DAC,150_, were collected at ϕ = −35°, −30°, −20°, −10°, 0° and 35° also for 12 h each.

Two data collections were carried out using crystals of l-arginine dihydrate. In the first, ambient pressure data were collected at 300 K using a crystal of 0.6 × 0.5 × 0.5 mm (referred to as **R**_300_). A total of ten patterns were collected for 2 h each using rotation steps of 20°.

A second crystal of 0.6 × 0.5 × 0.4 mm^3^ (referred to as **R**_DAC_) was loaded into the mini-DAC. Again, the applied pressure was low, *ca.* 0.25 GPa. In this experiment the gasket was Ti–Al–V (6% Al 4% V) alloy 1 mm thick, manufactured by laser cutting and contained a pre-drilled conical hole of 0.5 mm diameter. This gasket was indented around the pre-drilled hole before being widened with a vertically mounted drill to 0.7 mm in diameter. Previous tests had shown that gaskets of Ti–Al–V alloy produce a very low background in diffraction images. The data set consisted of 12 patterns collected for 12 h each at ϕ values of −30° to −10° in 5° steps, −85° to −95° in 5° steps, and 30° to 20° in 5° steps.

### 2.5. Indexing and processing of data collected at ambient pressure   

Ambient-pressure diffraction patterns (**A**_300_, **B**_300_, **B**_150_ and **R**_300_) were indexed and processed using the program *LaueG* (Piltz, 2015 ▸). Reflection intensities were integrated with a modified two-dimensional version of the minimum σ(*I*)/*I* algorithm formulated by Wilkinson *et al.* (1988 ▸) and Prince *et al.* (1997 ▸). The crystallographic resolution limit for integration was determined iteratively by finding the *d*-spacing at which *ca* 5% of integrated reflections had *I*/σ(*I*) ≥ 5.

Data were normalized to a single common incident wavelength by comparison of repeat observations and equivalent reflections with wavelengths within the range 0.85–3.5 Å using the program *LAUE*4 (Piltz, 2011 ▸). Due to the small size of the crystals no absorption or extinction corrections were applied. The crystal structures were refined against |*F*|^2^ using all data in *CRYSTALS* (Betteridge *et al.*, 2003 ▸). Since the neutron Laue method does not allow accurate empirical determination of the unit-cell volume, unit-cell dimensions were taken from literature values where available, otherwise values were calculated from a Berman thermal equation-of-state derived from X-ray powder measurements using *EOSFIT* 7.0 (Stevens & Hope, 1975 ▸; Angel *et al.*, 2014 ▸; Berman, 1988 ▸).

### 2.6. High-pressure data processing   

**Figure 4:**
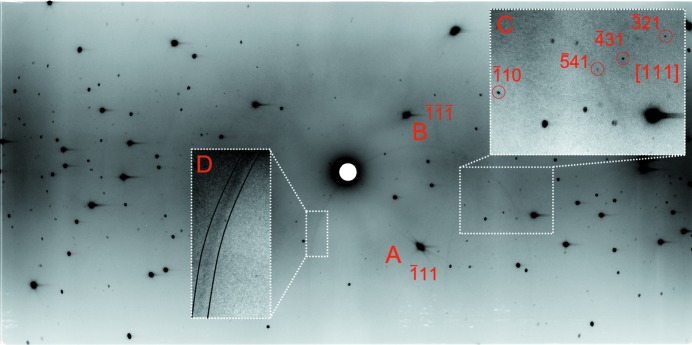
Laue diffraction pattern recorded at 150 K, ϕ = −30° using the mini-DAC. The pattern is dominated by scattering from the two diamond anvils: A marks the 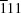 reflection of one anvil; B marks the 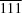 reflection of the other anvil. Inset C shows four reflections from the sample along the [111] zone, reflection 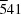 lies on top of a pseudo-Kossel line. Inset D highlights the same pseudo-Kossel line which is centred on the reflection marked by A. The contrast in each inset is adjusted to highlight certain features.

Processing the high-pressure data presented additional difficulties. The most troublesome features were the very intense reflections distributed throughout the pattern arising from the two diamond anvils (Fig. 4 ▸). These reflections were intense enough to produce a streaking effect on the read pattern due to long-lived fluorescence from the detector material as the detector cylinder was rotated during the reading process. Pseudo-Kossel lines (see above) were also observed around some diamond reflections. The fluorescence streaks and pseudo-Kossel lines produced localized areas of marginally increased background, complicating background modelling for sample reflections which straddled the edges of these features. The gasket material itself can produce spots, streaking or other background features depending upon composition and texture. Such features might be easily mistaken for sample reflections during the centring procedure described above.

#### 2.6.1. Indexing and orientation refinement from the high-pressure patterns   

The sample crystal was more than 500 times smaller than the total illuminated volume of the diamonds; consequently sample reflections were far weaker and had a smaller area than all but the weakest diamond reflections, and could therefore be distinguished in data-collection images.

The two sets of intense diamond peaks, typically 50–100 in number, were picked manually and indexed in *LaueG*. Orientation off-sets were refined for each pattern to account for the orbital rotation of the diamonds about the centred sample over the course of the experiment. The diamond reflections were then masked out and the remaining reflections used to index the sample, initially using strong reflections, but then iteratively including progressively weaker data until all sample reflections had been located and indexed. The orientation matrix obtained from analysis of one pattern was then applied to the other patterns by rotation about *z* and then re-refined for each pattern.

Comparison of the refined sample *xy*-offset values for the full set of patterns confirmed the sample had been centred to within 0.07 mm.

#### 2.6.2. Development of model spot profiles and integration   

Model spot profiles determined using intense sample spots were used to define the areas of integration of nearby weaker spots (Wilkinson *et al.*, 1988 ▸). Inaccurate model profiles arise if the data used for profile learning are contaminated by overlapping diamond reflections. Sample peaks in the region of the most intense diamond reflections were omitted on this basis. Reflections in the region of weaker diamond reflections were identified by cross-checking predicted sample- and diamond-reflection coordinates; peaks were considered overlapped if they lay within 10 pixels of each other (pixels are 0.2 × 0.2 mm^2^). The figure of 10 pixels was derived by trial and error and found to reject the weak (but relative to the sample, very intense) diamond peaks without rejecting a significant number of genuine sample reflections.

Integration of the high-pressure data was carried out pattern-by-pattern following a similar procedure as described above for the ambient-pressure data sets. Under ambient conditions there is no change in resolution with ϕ. With the high-pressure cell, the path of a ray through the cell body or diamonds is strongly dependent on ϕ and as a result the resolution limit (minimum observable *d*-spacing by the criterion defined in §2.5) changes with ϕ.

#### 2.6.3. Cell-body transmission   

In high-pressure single-crystal X-ray diffraction the detector is partially masked by the metallic body of the diamond-anvil cell, which leads to low completeness for all but high-symmetry cubic samples. The penetrating power of neutrons means that diffracted beams can pass through the small cell body to provide useable diffraction spots on the detector, greatly augmenting data from diffracted beams passing only through the diamond anvils. However, absorption corrections associated with these two classes of reflection are different, and it is necessary to take the difference into account during data reduction.

**Figure 5:**
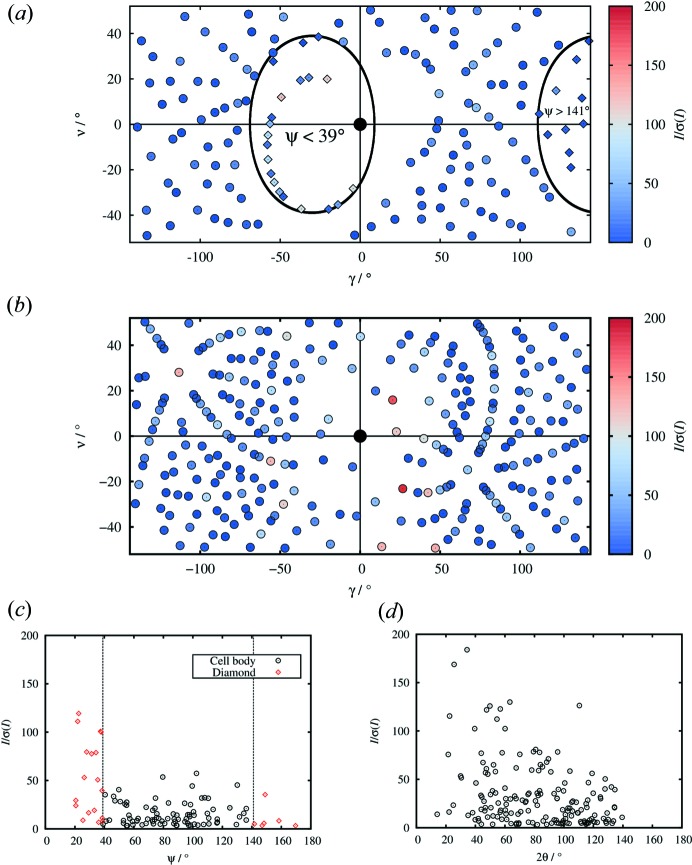
Simulated patterns for (*a*) **B**_150,DAC_ with ϕ = −30° and (*b*) **A**_300_. Reflections are coloured according to *I*/σ(*I*); reflections passing through the cell body in (*a*) are shown as circles, those passing through the diamonds are marked by diamonds. (*c*) A plot of *I*/σ(*I*) against scattering angle ψ for the pattern (*a*) for **B**_DAC,150_. Scattering angle ψ is used rather than 2θ to make a clear distinction between reflections passing through the diamonds (red diamonds) and the cell body (black circles). (*d*) Plot of *I*/σ(*I*) against 2θ for the pattern shown in (*b*) for **A**_300_.

Figs. 5 ▸(*a*) and (*b*) illustrate the distribution of reflections on the detector surface for one pattern of data set **B**_DAC,150_ (at ϕ = −30°, corresponding to the observed pattern shown in Fig. 4 ▸) and one of data-set **A**_300_. Reflection locations are expressed using the horizontal and vertical scattering angles γ and ν, and the magnitude of *I*/σ(*I*) is illustrated using colour. In Fig. 5 ▸(*a*) reflections passing through the diamonds (with (ψ < 39° or ψ > 141°) are shown as diamonds, and those passing through the cell body (39 < ψ < 141°) as circles. The black ellipse and half-ellipse mark the boundaries between these two types of reflection; these are centred at 30° and 150° because ϕ = −30° for this image. Figs. 5 ▸(*c*) and (*d*) show the distributions of *I*/σ(*I*). In Fig. 5 ▸(*c*), values are plotted against scattering angle, ψ, rather than 2θ to distinguish between reflections passing through the diamonds and the cell body.

The maximum *I*/σ(*I*) values of **B**_DAC,150_ are lower than those of **A**_300_ reflecting the small sample size, and attenuation of the incident and scattered beams by the mini-DAC. The average *I*/σ(*I*) values for the patterns above are 15.9 for the 157 reflections in the **B**_DAC,150_ pattern and 20.4 for the 273 reflections in the **A**_300_ pattern. Attenuation of the diffracted beams and the higher background due to scattering from the cell accounts for the 25% reduction in average *I*/σ(*I*).

Of the 157 reflections in Fig. 5 ▸(*a*) 30 (19%) pass only through the diamonds. The very low neutron absorption cross section of diamond (σ_abs_ = 0.0035 barn) compared with that of the cell-body (Be–Cu alloy, *ca* 99.5% Cu, σ_abs_ = 3.78 barn for Cu, σ_abs_ = 0.0076 barn for Be) means that these reflections are essentially unattenuated. However, the data in Figs. 5 ▸(*a*) and (*c*) indicate that it is possible to collect reflections over the whole image plate, not just in the areas where reflections pass only through the diamonds, without prohibitive reductions in data quality (*cf.* Figs. 5 ▸ *c* and *d*). Some ‘back-scattered’ reflections pass through the diamond on the incident-beam side of the mini-DAC, visible at γ > 120° and are recorded at ψ > 141° (Figs. 5 ▸ *a* and *c*, respectively). This is particularly advantageous, as these reflections are typically high resolution and composed of a single wavelength. When the cell axis is aligned with the incident beam (*i.e.* in the setting with ϕ = 0) these reflections are masked by the erasing lamps and casings.

The majority of the most intense reflections in Figs. 5 ▸(*a*) and (*b*) are harmonically overlapped and, although they are used for defining integration model profiles, they are omitted from the final intensity data set used for structure analysis.

#### 2.6.4. Correction for cell attenuation   

The degree of attenuation of a diffracted beam is dependent upon the path length through the cell, the materials encountered *en route* to the detector, and the wavelength of the reflection (attenuation increases with wavelength). For each reflection the path to the detector is characterized by a set of ψ- and ϕ-dependent angles. A set of angular limits were derived from the dimensions of the mini-DAC, dividing the mini-DAC into a series of zones for which the path lengths through the gasket, diamonds and cell-body can be determined and an attenuation factor defined. Details are available in the supporting information. The wavelength-dependent linear attenuation coefficients of diamond, steel or beryllium–copper (Be–Cu) alloy were calculated from the chemical composition and density using the NIST Neutron Attenuation and Activation tool (NIST, 2005 ▸). Since the linear attenuation of diamond is approximately 300 times smaller than that of Be–Cu, the attenuation by the anvils was neglected for all reflections with ψ < ω.

**Figure 6:**
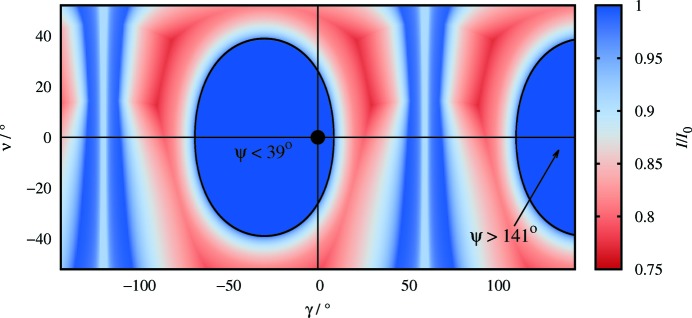
The attenuation as calculated for λ = 1.3 Å pixel-by-pixel at ϕ = −30°, corresponding to Figs. 4 ▸ and 5 ▸(*a*).

The most intense wavelength in the KOALA spectrum is 1.3 Å and for this wavelength there is no region of the detector plate for which the attenuation factor *I*/*I*_0_ is less than 0.75, and *I*/*I*_0_ > 0.9 for around 60% of the detector. These figures are wavelength specific; attenuation is greater for longer wavelengths and lower for shorter wavelengths. For example, in both data sets **B**_DAC,300_ and **B**_DAC,150_, approximately 90% of the sample reflections occur at wavelengths less than 2.4 Å; at this approximate upper limit no region of the image plate exhibits *I*/*I*_0_ < 0.65, with *I*/*I*_0_ > 0.8 for around 60% of the detector area. The distribution of attenuation at λ = 1.3 Å over the image plate is shown in Fig. 6 ▸ for the cell rotated to ϕ = −30° for comparison with Figs. 4 and Fig. 5 ▸(*a*).

Attenuation in the upper half of Fig. 6 ▸ is greater than in the lower half because the cell is triangular and mounted with its widest section uppermost (Fig. 3 ▸ *a*). The vertical areas where *I*/*I*_0_ ≃ 1.0 in Fig. 6 ▸ around −120° and 60° correspond to beam paths exiting through the side of the cell between the two halves of the cell body, and for the narrow range *I*/*I*_0_ < 1.0, through the gasket.

There are two additional attenuation processes that can occur as a result of the mini-DAC for which corrections were not applied. First, the diamonds diffract intensity away from diffracted sample rays that pass through them if the Bragg condition is met for the wavelength of the diffracted ray. This effect is referred to as a *diffracted-beam event*. Secondly, attenuation arises in the incident beam due to the reactor-side diamond diffracting significant intensity away from the sample, an effect referred to as an *incident-beam event* or *diamond dip*. Diamond dips have been examined for monochromatic X-ray diffraction from both laboratory and synchrotron sources, and in the monochromatic case occurs when the diamonds and sample diffract in the same setting (Loveday *et al.*, 1990 ▸). In Laue diffraction, intensity is integrated over wavelength rather than angle of sample rotation, nevertheless such incident- or diffracted-beam events will only occur for a particular sample reflection if the Bragg condition is met by a diamond anvil for the wavelength of the sample reflection. Since the beam divergence in the neutron Laue experiment is usually smaller than the beam divergence in the monochromatic experiment, the likelihood of an event occurring for a particular sample reflection is lower, although the reduction in the integrated observed intensity when an event occurs will be higher. For the complete data set, the mean effect of incident- and diffracted-beam events on the observed sample intensities will be the same for the Laue and monochromatic experiments on the same sample and pressure cell.

Wavelength overlaps between sample and diamond reflections were calculated for the five strongest classes of diamond reflections: 111, 220, 311, 400 and 440 pattern-by-pattern. The overlaps were checked against the list of outliers for the merged dataset. During merging and normalization (see §2.6.5), a reflection was rejected as an outlier if the difference between reflection intensity *I* and average reflection intensity 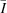 was more than 10σ(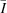). Of the outliers in the normalized data for **B**_DAC,150_ and **B**_DAC,300_, less than 10% overlapped with a strong diamond reflection suggesting that the effect of incident-beam events is largely removed during the normalization procedure.

#### 2.6.5. Normalization   

Since the Bragg condition is met for many reflections over the diffraction volume at various wavelengths during the experiment, it is necessary to scale the intensities to one common wavelength for refinement. A reflection (and its symmetry equivalents) can be measured at several wavelengths and the diffracted intensity is dependent on the wavelength spectrum of the instrument. The normalization routine in *LAUE4* scales the intensities of measured reflections to a common reference using a least-squares fit to this distribution, the resulting refined incident-beam spectrum can then be used as the basis for a second iteration, improving the merging statistics.

For ambient pressure data, redundancy is typically high enough that the spectrum accepted can be reduced to the most intense band at 0.85–1.7 Å, however, the high-pressure data sets contain fewer well measured reflections and so a wider wavelength spectrum from 0.8–3.5 Å is utilized. This introduces weaker, long-wavelength data resulting in increased *R*_merge_ values, but increases the number of reflections for refinement.

Due to the various attenuations in incident and diffracted beams described above, the effective incident-beam spectrum is significantly different for samples within the mini-DAC. When constrained to the nominal incident-beam spectrum, the normalized reflection intensities are under- or over-inflated to match the spectrum exactly. Allowing the incident beam spectrum to refine removes this constraint and allows the normalization procedure to remove data affected by incident- and diffracted-beam events throughout the data set.

## 3. Results   

### 3.1. Ambient-pressure neutron diffraction of HMT   

While crystal **A** was large by X-ray standards, it is on the lower limit for usable samples on KOALA. For **A**_300_ the resolution limit (§2.5) was 0.72 Å, giving a data set with 〈*I*/σ(*I*)〉 = 59.62; the value of 〈*I*/σ(*I*)〉 to a resolution of 1 Å was 119.38. Diffraction data for crystal **B**, which had a volume approximately five times smaller than Crystal **A**, extended to 1.04 Å with 〈*I*/σ(*I*)〉 to 46.76. Cooling substantially improves the statistics for **B**, and at 150 K (data-set **B**_150_) data could be integrated to 0.77 Å with 〈*I*/σ(*I*)〉 = 36.99; 〈*I*/σ(*I*)〉 to 1.0 Å was 63.90. Data quality statistics are given for all data sets in Table 1 ▸. Completeness values for Laue diffraction cannot reach beyond the theoretical maximum of 83.3% due to harmonic overlap (Cruickshank *et al.*, 1987 ▸, 1991 ▸).

The atomic coordinates used to initiate refinement were taken from the Cambridge Database (Allen, 2002 ▸) entry HXMTAM07 (Terpstra *et al.*, 1993 ▸). For **A**_300_ all atoms were refined anisotropically without restraints giving a final *R*-factor (*R*1[*F* > 4σ(*F*)]) of 0.0368. However, data set **B**_300_ at the same temperature consisted of just 20 unique reflections and unrestrained refinement was limited to an isotropic treatment for all atoms, giving a data/parameter ratio of 2.5. In comparing data/parameter ratios it should be noted that H-atom parameters are refined, unlike most refinements against X-ray data. The *R*-factor for the free refinement was 0.0608. The effective data-to-parameter ratio for anisotropic refinement was increased by applying shift-limiting restraints to anisotropic displacement parameters (ADPs), as well as rigid-body and rigid-bond restraints to the C and N atoms. This stabilized a fully anisotropic refinement of all atoms, yielding a final *R*-factor of 0.0272.

The 150 K data-set (**B**_150_) consisted of 41 unique data, and the model was freely refined with anisotropic displacement parameters for all atoms, yielding *R* = 0.0346 with a data/parameter ratio of 2.9.

### 3.2. Diamond-anvil cell data for HMT   

The most remarkable result from this study is that there is a no significant reduction in completeness for data collected from the mini-DAC (Table 1 ▸). In contrast to high-pressure X-ray diffraction, the use of neutrons, which are highly penetrating and can pass through the cell body, allows the observation and incorporation of data without angular restriction. This is particularly advantageous for low-symmetry crystals where the restriction for X-rays leads to very low completeness values; by contrast Laue diffraction is subject to a theoretical maximum completeness of 83.3% regardless of crystal symmetry, and only marginally affected by the use of a high-pressure cell.

Data collected from crystal **B** in the mini-DAC at 300 K (**B**_DAC,300_) on KOALA were integrated to a resolution range of 0.90–1.04 Å (the maximum resolution obtained for each image depending on the orientation of the cell, see above). Given the low number of unique reflections (19), the unrestrained refinement was limited to an isotropic model giving a final *R*-factor of 0.0590. Use of the restraints described above for anisotropic refinement yields *R* = 0.0388.

The same integration procedure was applied to **B**_DAC,150_ resulting in a significant improvement in resolution range to 0.79–0.92 Å. At this temperature the data set consisting of 35 unique reflections was used to refine a fully anisotropic model with rigid-body and rigid-bond restraints applied to C and N atoms only, the final *R*-factor was 0.0762. For comparison, a freely refined isotropic model gave *R* = 0.0797.

The larger *R*-factor for **B**_DAC,150_ compared to **B**_DAC,300_ reflects the inclusion of numerous weaker high-resolution data. At room temperature weaker data are either absent or rejected as outliers during normalization. Agreement factors for structures determined by neutron Laue diffraction are usually higher than those determined by monochromatic techniques, and the magnitude of the standard uncertainties of the structural parameters provides a more robust indication of the structure quality (McIntyre *et al.*, 2006 ▸). The *R*-factors for **B**_300_ and **B**_DAC,300_ are in fact unusually low, reflecting the almost complete absence of weak data.

**Figure 7:**
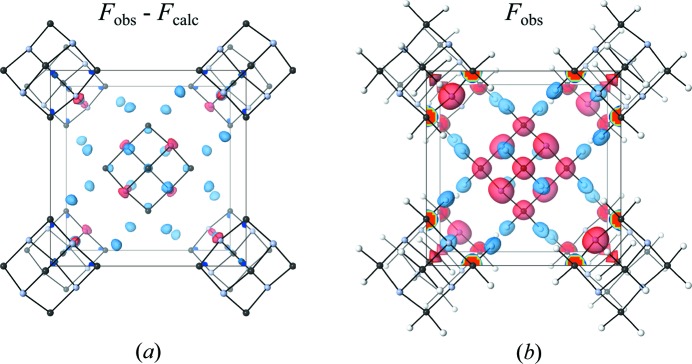
Isosurface plots of (*a*) **B**_DAC,150_ Fourier difference map for a model phased with just the C and N positions. Strong negative scattering density can be seen at the H atom position, along with other residual density. Isosurface levels are at ±4 fm Å^−3^. (*b*) Observed scattering density for **B**_DAC,150_ clearly showing the negative scattering density associated with the H atoms, isosurface levels are at ±9 fm Å^−3^. Positive scattering density is shown in red, negative scattering density in blue. Images were generated in *VESTA* 3 (Momma & Izumi, 2011 ▸).

Location of H-atom positions is one of the principal uses of neutron diffraction. Fig. 7 ▸ shows the (*a*) *F*_obs_ − *F*_calc_ and (*b*) *F*_obs_ scattering density isosurfaces for **B**_DAC,150_. For a model without H atoms, the difference map clearly identifies the absent H-atom position, and since the sample is undeuterated, the increased contrast of the strong negative scattering length of H aids this identification.

### 3.3. Validation of the refined structural parameters of HMT   

HMT has not been studied previously by neutron diffraction at 150 K so comparison was made to a reported structure at 160 K by Kampermann *et al.* (1995 ▸). Room-temperature data are compared to the refinements of Terpstra *et al.* (1993 ▸). Bond lengths and angles of all structures are listed in Table 2 ▸.

**A**_300_ reproduces the literature bond distances to within 3 standard deviations with the largest discrepancy being in the C—H bond, elongated by 0.031 (8) Å. The reduction in sample volume between **A**_300_ and **B**_300_ introduced a significant discrepancy of 0.08 (1) in C—H bond length compared with the literature value, while the C—N distance was reproduced accurately. It appears that these discrepancies arise from the short counting times of just 4 h per pattern for both crystals. Counting time was increased to 12 h for **B**_150_, as a result all bond lengths and angles for **B**_150_ were within 1.5 standard deviations of literature values. Restraining the bond distances to the literature values increases the *R*-factor for **B**_150_ to 0.0364 (+0.18%) and for **B**_300_ the *R*-factor increases to 0.0382 (+1.1%).

**Figure 8:**
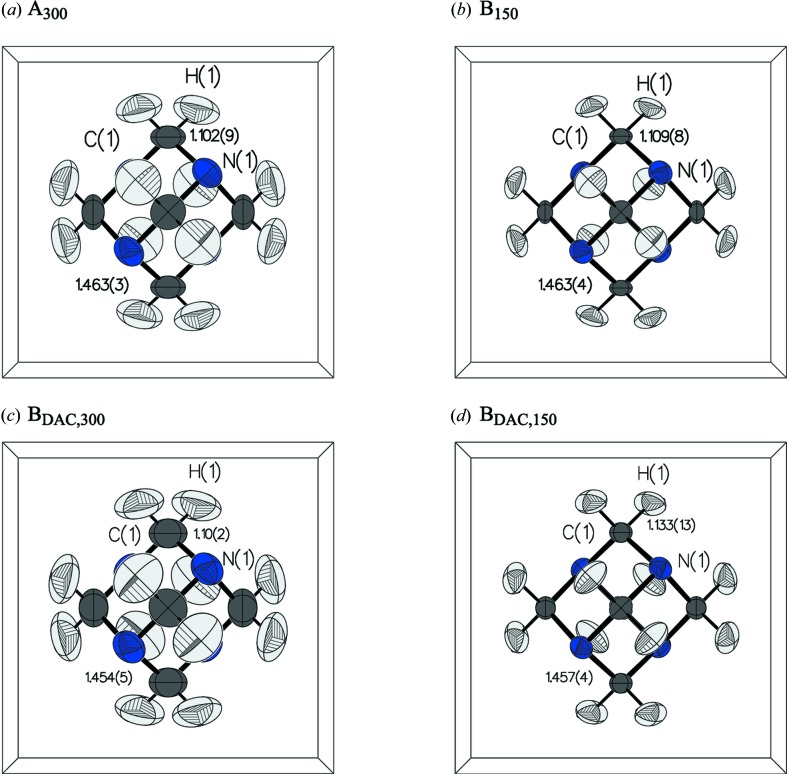
Summary of refined HMT structures illustrating bond distances. (*a*) Room-temperature, ambient-pressure structure of **A**_300_. (*b*) 150 K, ambient-pressure structure of **B**_150_. (*c*) Room-temperature, high-pressure structure of **B**_DAC,300_. (*d*) 150 K, high-pressure structure of **B**_DAC,150_.

For data set **B**_DAC,300_ both C—N and C—H bond distances were statistically equal to the literature values. Likewise, **B**_DAC,150_ closely reproduced the literature data at 160 K, with all structural parameters within one standard deviation. Despite the higher *R*-factor, the refined structure for **B**_DAC,150_ shows estimated standard deviations that are very similar to those of **B**_150_. A graphical summary of the refined structures under various conditions is given in Fig. 8 ▸; crystallographic data for each refined structure are given in Table 3 ▸.

### 3.4. Analysis of anisotropic displacement parameters of HMT   

Both data sets collected at ambient conditions, **A**_300_ and **B**_300_, show similar ADP parameters. Although the C atom in **B**_300_ is slightly more oblate, (*U*_3_/*U*_1_ = 3.99 *versus* 2.57 for **A**_300_) *U*_eq_ values for all atoms within one estimated standard deviation of each other.

For data sets **B**_150_ and **B**_DAC,150_ there is no statistically significant difference between *U*_eq_ parameters for C, N and H atoms and both H atoms show very similar *U*_3_/*U*_1_ values, with *U*_3_/*U*_1_ = 3.69 *versus* 3.45 at ambient pressure.

In refinements at both ambient and high pressure, where the H ADPs were freely refined, there was no significant difference in mean-square displacement along the C—H bonds (*i.e.* 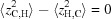) implying that the H ADPs pass the Hirshfeld rigid-bond test (Hirshfeld, 1976 ▸; Eriksson & Hermansson, 1983 ▸). Displacement distances, *U*_3_/*U*_1_, and *U*_eq_ values for all data sets are given in Table 4 ▸.

## 4. l-Arginine dihydrate   

Data for crystal **R**_300_ could be integrated to a resolution of 0.96 Å, giving a data set with 〈*I*/σ(*I*)〉 = 20.26, increasing to 23.11 at 1.0 Å. For the crystal placed inside the cell, **R**_DAC_, the integration limit was also 0.96 Å, with 〈*I*/σ(*I*)〉 = 25.37, increasing to 27.07 at a resolution of 1.0 Å. Most importantly, as can be seen in Table 5 ▸, completeness values are not affected by placing the sample inside the mini-DAC. The increased redundancy and 〈*I*/σ(*I*)〉 values for **R**_DAC_ are due to the much longer exposure time for the sample in the DAC.

**Figure 9:**
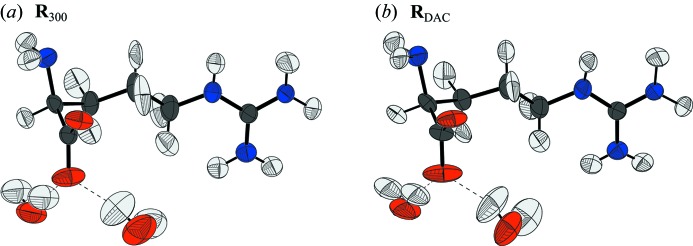
Refined structures of l-arginine dihydrate from data collected under (*a*) normal conditions and (*b*) within the mini-DAC.

Refinement of both data sets required the use of rigid-body and rigid-bond restraints applied to all atoms. In addition, bond-angle and bond-distance restraints were applied to both water molecules. Combined these restraints help alleviate the low data-to-parameter ratios for both data sets: 1.61 for **R**_300_ and 1.68 for **R**_DAC_. The resulting agreement factors are *R* = 0.0750 for **R**_300_, and *R* = 0.1011 for **R**_DAC_, the final refined asymmetric units are shown in Fig. 9 ▸.

Comparison of geometric parameters between **R**_300_ and **R**_DAC_ structures shows that all bond lengths are reproduced in the DAC data within statistical significance and of the 29 interatomic bonds, 25 show differences less than three standard deviations. Likewise, all intermolecular contacts are reproduced within statistical significance.

## 5. Conclusions   

We have shown that high-pressure single-crystal neutron diffraction data can be collected from a sample in the miniature DAC with no significant reductions in completeness or resolution compared with equivalent data collected at ambient pressure. The data are of similar quality, as judged by *R*-factors, geometric parameters and estimated standard deviations, to those obtained at ambient pressures without the mini-DAC. This is achieved mainly by the ability to measure diffracted intensity directly through the body of the mini-DAC, the geometric simplicity of the mini-DAC facilitating the derivation of the necessary attenuation corrections. Except for miniaturization and the material used for its construction, the DAC used in this study is identical to those used for conventional high-pressure X-ray diffraction and spectroscopic measurements, so that a full set of characterization data can be obtained under precisely the same conditions with a single sample loading. The cell would also be suitable for gas-loading techniques.

There are certain experimental difficulties associated with using the miniature DAC. The centring process takes a significant proportion of experiment time, which is an important consideration when beam time is limited. The procedure also relies on successfully identifying sample spots in the short 1 h exposures used during this process; centring by maximizing the intensities of the diamond or gasket reflections mis-centres the sample and leads to unusable data. Determining the orientation of the sample within the DAC by X-ray diffraction prior to the neutron experiment would allow spot positions to be predicted and cross-checked to avoid this pitfall.

The high-pressure methods described are also suitable for low-temperature high-pressure experiments where the improved diffraction quality counter-balances the continuing use of small crystals, particularly of high symmetry. The cryofurnace on the KOALA instrument allows the entire cell to be cooled easily, although changes in the cell pressure caused by cooling cannot currently be probed by on-line measurement due to the lack of optical access and the intrinsic limitation of the Laue technique that unit-cell dimensions cannot be known absolutely, only the ratios of *a*:*b*:*c*. It should be noted that facilities for carrying out identical low-temperature high-pressure experiments using X-rays are also still far from routine (Ridley & Kamenev, 2014 ▸).

For opposed-anvil pressure cells, the cell generates a load which is converted to pressure applied to the sample. The magnitude of this pressure is determined by the combination of culet size, and gasket dimensions and material. The loads generated by the mini-DAC are equal to those generated by standard Merrill–Bassett type cells which are routinely capable of reaching 20–25 GPa with 600 µm culets and 250 µm gasket holes. The pressure limit for neutron experiments with the mini-DAC will thus be somewhat reduced by the need to use a larger sample crystal and therefore larger culets and gasket holes, and the upper pressure limit for the current culet and gasket dimensions is estimated to be 5 GPa.

A trial of a panoramic moissanite-anvil cell from the Geophysical Lab was carried out on the VIVALDI diffractometer at ILL using a 1 × 1 × 0.5 mm crystal of natrolite (Xu *et al.*, 2002 ▸; McIntyre *et al.*, 2005 ▸). This cell design has two advantages over the miniature DAC; firstly that the illuminated volume of the anvils is significantly reduced by directing the beam perpendicular to the cell axis through the gasket; as a result the contaminating anvil reflections are reduced in number and intensity. Also the strongly supported anvil design allows large samples to be taken well beyond the pressure limits of the miniature DAC. However, the optical access afforded by this design would prohibit joint X-ray–neutron studies of the type envisaged for the miniature DAC.

Despite the limitations imposed on the crystal volume, fully anisotropic refinements resulted in C—N and C—H bond lengths within experimental error of the benchmark neutron literature values for the high-symmetry hexamine structure. Further experiments using l-arginine dihydrate demonstrate that the benefits of high completeness extend to more complex, lower-symmetry structures. The way is now open towards X-ray and neutron diffraction studies of more complex systems at high pressures, giving the capability to perform joint diffraction studies under the same conditions on the same crystal.

## Supplementary Material

Attenuation correction procedure. DOI: 10.1107/S2052252516000725/fs5125sup1.pdf

## Figures and Tables

**Table 1:** Crystallographic statistics for all **HMT** data sets

Data set	Resolution (Å)	*R*_merge_	Completeness (%)	Redundancy	〈*I*/σ(*I*)〉 (to 1 Å)	Total no. of reflections
**A**_300_	0.72	0.043	74.2	19.8	59.62 (119.38)	997
**B**_300_	1.04	0.103	74.1	17.4	46.76	475
**B**_150_	0.77	0.108	73.2	15.2	36.99 (63.90)	850
**B**_DAC,300_	1.04	0.146	70.4	24.8	59.13	670
**B**_DAC,150_	0.79	0.195	67.9	16.6	32.30 (39.83)	878

**Table 2:** Bond distances (Å) and angles (°) for **HMT** data collected at ambient pressure and using the mini-DAC with reference literature values

	Literature data						
Bond length or angle	300 K^a^	160 K^*b*^	**A**_300_	**B**_300_ †	**B**_150_	**B**_DAC,300_ †	**B**_DAC,150_ †
N—C	1.462 (5)	1.4660 (15)	1.462 (3)	1.462 (3)	1.463 (4)	1.454 (5)	1.457 (4)
C—H	1.071 (6)	1.097 (3)	1.102 (8)	1.148 (8)	1.109 (8)	1.10 (2)	1.133 (13)
C—N—C	108.0 (3)	107.89 (9)	107.78 (16)	107.08 (17)	108.0 (2)	107.6 (3)	107.6 (4)
N—C—N	112.4 (3)	112.54 (9)	112.8 (3)	114.1 (3)	112.4 (4)	113.0 (5)	113.1 (7)
N—C—H	108.1 (3)	108.46 (16)	108.3 (4)	107.8 (4)	108.6 (4)	108.6 (4)	108.7 (3)
H—C—H	112.2 (6)	110.5 (2)	110.8 (7)	111.7 (6)	109.9 (7)	109.5 (15)	108.8 (9)

References: (*a*) Terpstra *et al.* (1993 ▸); (*b*) Kampermann *et al.* (1995 ▸).

† Values are derived from restrained anisotropic refinements.

**Table 3:** Crystallographic data for ambient and high-pressure structures of HMT

Data set	**A**_300_, ambient pressure	**B**_300_, ambient pressure	**B**_150_, ambient pressure	**B**_DAC,300_, pressure cell	**B**_DAC,150_, pressure cell
Crystal data					
Chemical formula	C_6_H_12_N_4_	C_6_H_12_N_4_	C_6_H_12_N_4_	C_6_H_12_N_4_	C_6_H_12_N_4_
Formula weight	140.19	140.19	140.19	140.19	140.19
Crystal system, space group	Cubic, 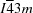	Cubic, 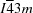	Cubic, 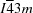	Cubic, 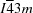	Cubic, 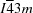
*a* (Å)	7.028 (2)	7.028 (2)	6.963 (4)	7.028 (2)	6.963 (4)
*V* (Å^3^)	347.1 (3)	347.1 (3)	337.6 (6)	347.1 (3)	337.6 (6)
*Z*	2	2	2	2	2
*D*_calc_ (g cm^−3^)	1.341	1.341	1.379	1.341	1.379
Crystal size (mm)	0.4 × 0.4 × 0.3	0.15 × 0.20 × 0.30	0.15 × 0.20 × 0.30	0.15 × 0.20 × 0.30	0.15 × 0.20 × 0.30
					
Data collection					
Temperature (K)	300	300	150	300	150
Pressure (GPa)	Ambient	Ambient	Ambient	Estimated 0.25	Estimated 0.25
Radiation (Å)	Neutrons 0.8–3.5	Neutrons 0.8–3.5	Neutrons 0.8–3.5	Neutrons 0.8–3.5	Neutrons 0.8–3.5
Range of *h*, *k*, *l*	*h* = −7 → 8, *k* = −9 → 8, *l* = −9 → 2	*h* = −6 → 6, *k* = −6 → 6, *l* = −2 → 6	*h* = −9 → 2, *k* = −6 → 7, *l* = −9 → 7	*h* = −6 → 4, *k* = −4 → 6, *l* = −5 → 6	*h* = −8 → 5, *k* = −5 → 7, *l* = −6 → 8
Total, unique data, *R*_int_	997, 81, 0.043	475, 30, 0.103	850, 66, 0.108	670, 29, 0.146	878, 57, 0.195
Observed data [*I* > 2.0 *σ*(*I*)]	71	30	55	30	46
					
Refinement					
*N*_ref_, *N*_par_, *N*_ref_/*N*_par_	49, 14, 3.5	20, 14, 1.43	41, 14, 2.93	19, 14, 1.36	35, 14, 2.5
(*R*[*F* ^2^ > 2*σ*(*F* ^2^)]), *wR*_2_, *S*	0.0368, 0.0956, 0.92	0.0272, 0.0757, 1.02	0.0346, 0.0881, 0.98	0.0388, 0.1039, 1.50	0.0762, 0.3292, 1.12
Δρ_min_, Δρ_max_ (fm Å^−3^)	−0.41, 0.46	−0.35, 0.43	−1.50, 1.07	−0.28, 0.33	−2.01, 3.30

**Table 4:** Displacement distances, *U*_3_/*U*_1_ and *U*_eq_ values for refined structures of HMT Values are derived from the restrained anisotropic refinements.

	*U*_eq_			*U*_3_/*U*_1_			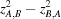	
Data set	N	C	H	N	C	H	N—C	C—H
**A**_300_	0.0466 (7)	0.0516 (11)	0.088 (3)	2.05	2.57	3.2	0.002 (2)	0.017 (5)
**B**_300_	0.0469 (6)	0.0503 (12)	0.0907 (14)	2.10	3.99	2.25	0.000 (2)†	0.015 (3)
**B**_150_	0.0285 (8)	0.0220 (11)	0.043 (2)	1.40	2.42	3.45	0.007 (2)	0.005 (4)
**B**_DAC,300_	0.0545 (8)	0.0653 (14)	0.1017 (19)	1.95	1.89	3.86	0.000 (3)†	0.007 (4)
**B**_DAC,150_	0.0262 (9)	0.0243 (13)	0.0463 (17)	1.43	1.56	3.69	0.000 (3)†	0.001 (4)

† Value restrained to 0.

**Table 5:** Crystallographic statistics for L-arginine dihydrate data sets

Data set	Resolution (Å)	*R*_merge_	Completeness (%)	Redundancy	〈*I*/σ(*I*)〉 (to 1 Å)	Total no. of reflections
**R**_300_	0.96	0.112	77.0	16.9	20.26 (23.11)	7872
**R**_DAC_	0.96	0.193	77.1	18.4	25.37 (27.07)	8926
