# Supplementary material for: Use of a miniature diamond-anvil cell in high-pressure single-crystal neutron Laue diffraction
Source: IUCrJ. 2016 Feb 26;3(Pt 3):168–79. doi: 10.1107/S2052252516000725 (PMC4856139; doi:10.1107/S2052252516000725)
Supplement: Supplementary file 1 [file m-03-00168-sup1.pdf]

Supporting information for: Use of a miniature diamond anvil cell in high-pressure single-crystal neutron Laue diffraction

Authors

Jack Binns[ab], Garry J McIntyre[a]*, Konstantin V Kamenev[c], Stephen A Moggach[b] and Simon Parsons[b]*

[a] Australian Nuclear Science and Technology Organisation, New Illawarra Road, Lucas Heights, NSW 2234, Australia

[b]EaStCHEM School of Chemistry and Centre for Science at Extreme Conditions, The University of Edinburgh, The King's Buildings, West Mains Road, Edinburgh, EH9 3FJ, United Kingdom

[c]School of Engineering and Centre for Science at Extreme Conditions, The University of Edinburgh, Erskine Williamson Building, The King's Buildings, Peter Guthrie Tait Road, Edinburgh, EH9 3JJ, United Kingdom

Correspondence email: gmi@ansto.gov.au; s.parsons@ed.ac.uk

Supporting information

S1. Attenuation correction calculation

The path length of a ray passing through the cell-body was determined in a reference axis system attached to the cell where $\mathbf{y}_{\text{cell}}$ lies along the cell axis, $\mathbf{z}_{\text{cell}} \mid\mid \mathbf{z}$, and $\mathbf{x}_{\text{cell}} = \mathbf{y}_{\text{cell}} \times \mathbf{z}_{\text{cell}}$. The cell has three-fold symmetry about $\mathbf{y}_{\text{cell}}$ and can be divided into six sectors based on the angle, χ_i, between the projection of a reflection $\mathbf{h}_i$ in the $\mathbf{xz}_{\text{cell}}$ plane of the cell reference system and the $\mathbf{z}_{\text{cell}}$ axis (Figure S1).

IMPORTANT: this document contains embedded data - to preserve data integrity, please ensure where possible that the IUCr Word tools (available from http://journals.iucr.org/services/docxtemplate/) are installed when editing this document.

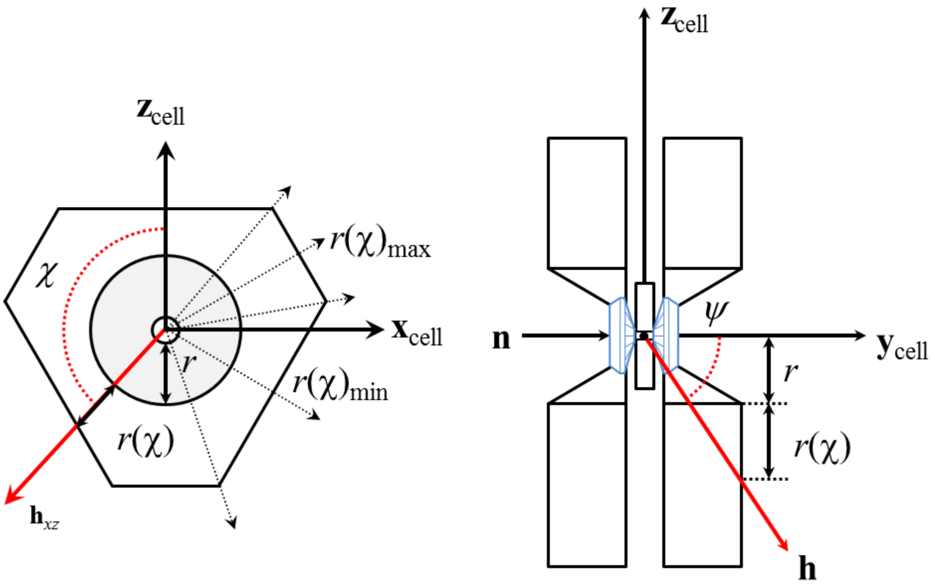

Figure S1 (Left) The mini-DAC has three-fold rotational symmetry allowing six sectors to be defined on the basis of angle χ (Right) The value of r(χ) is used to define a further set of sectors creating limiting values of ψ. Within each sector a trigonometric expression determines the total path length through the cell body. The thickness of the gasket and sample is exaggerated for clarity.

For the cell rotated to an angle ϕ, the cell axis system of unit vectors is determined by applying rotation matrix **Φ** as in 1 for $\mathbf{x}_{cell}$.

$$\mathbf{x}_{cell} = \mathbf{\Phi} \cdot \mathbf{x} = \begin{pmatrix} \cos\phi & \sin\phi & 0 \\ -\sin\phi & \cos\phi & 0 \\ 0 & 0 & 1 \end{pmatrix} \cdot \begin{pmatrix} 1 \\ 0 \\ 0 \end{pmatrix} \quad 1$$

The model of the cell is constructed from a χ-independent section denoted *r* (5.25 mm) and a χ-dependent length, *r*(χ), to account for the change in cell shape with rotation about $\mathbf{y}_{cell}$ between upper and lower limits of 6.25 and 3.25 mm. For each reflection, the detector coordinates (x_i, z_i) and radius *R*, determine the ray $\mathbf{h}_i$ by equations 2 and 3.

$$\gamma = \frac{x_i}{R} \quad 2$$

$$\mathbf{h}_i = \begin{pmatrix} R/\sin\gamma \\ R/\cos\gamma \\ z_i \end{pmatrix} \quad 3$$

The angles χ_i and ψ_i and consequently value of $r(\chi_i)$:

$$\chi_i = \operatorname{acos}\left(\frac{\mathbf{h}_{xz,i} \cdot \mathbf{z}_{cell}}{|\mathbf{h}_{xz,i}||\mathbf{z}_{cell}|}\right) \quad 4$$

$$\psi_i = \operatorname{acos}\left(\frac{\mathbf{h}_i \cdot \mathbf{y}_{cell}}{|\mathbf{h}_i||\mathbf{y}_{cell}|}\right) \quad 5$$

For example, in the range 0° < χ < 45° $r(\chi)$ is given by A6, $r(\chi)_{min}$ is determined at 0°, $r(\chi)_{max}$ at 60°. The other limiting angles are χ = 75°, 120°, and 165° between which similar expressions are used with $r(\chi)_{min}$ or $r(\chi)_{max}$ as appropriate.

$$r(\chi_i) = \frac{r + r(\chi)_{min}}{\cos \chi_i} - r \quad 6$$

For the backing-plate thickness, cell body separation, gasket dimensions, and $r(\chi_i)$, a set of limiting ψ_i angles are calculated to determine whether the diffracted ray passes through the diamonds, gasket, and cell body.

For rays passing through the cell body, the path length is determined by calculating points of intersection with the cell faces. Path lengths through the gasket are calculated by a simple trigonometric expression. Once the path lengths through the cell body $l_{i,cb}$, diamonds $l_{i,d}$, and gasket $l_{i,g}$, are determined, the attenuation for each reflection i is then calculated using the expression 7. Since the attenuation due to the diamonds is so low relative to the gasket and cell body, this term is omitted for $\psi > \omega$. The guide pins (Cu-Be) and screws (Fe) are omitted from the model. The gasket was circular in shape simplifying the correction term. Details are given in Table S1.

$$a_i(\psi_i, \chi_i, \lambda_i) = \frac{I}{I_0} = e^{-\mu_{cb}(\lambda_i) l_{i,cb}(\psi_i,\chi_i)} e^{-\mu_g(\lambda_i) l_{i,g}(\psi_i,\chi_i)} e^{-\mu_d(\lambda_i) l_{i,d}(\psi_i,\chi_i)} \quad 7$$

Table S1 Expressions for determining angular limits and path length l_i. Calculations are carried out in the plane containg the diffracted beam h and the cell axis y_{cell}. b_t is the cell body thickness (5 mm), b_s is half the cell body separation (1.625 mm), r is the fixed cell radius (5.25 mm) which is the sum of the radius of the cell opening d_r (2 mm) and the inner cone radius r_{cone} (3.25 mm), $r(\chi)$ is the χ-dependent cell radius (maximum value 6.25 mm, minimum value 3.25), g_t is half the gasket thickness, g_h is the gasket hole radius and g_r is the gasket radius. The diamond height, d_h is 3 mm, set into the body by 1.5 mm, d_r is the radius of the cell opening (2 mm).

**ψ limits for cell body**	
$\omega = \mathrm{atan}\left(\frac{r}{b_t + b_s}\right)$	Cell-opening angle.
$\psi_1 = \mathrm{atan}\left(\frac{r + r(\chi)}{b_s}\right)$	High-angle limit of cell body.

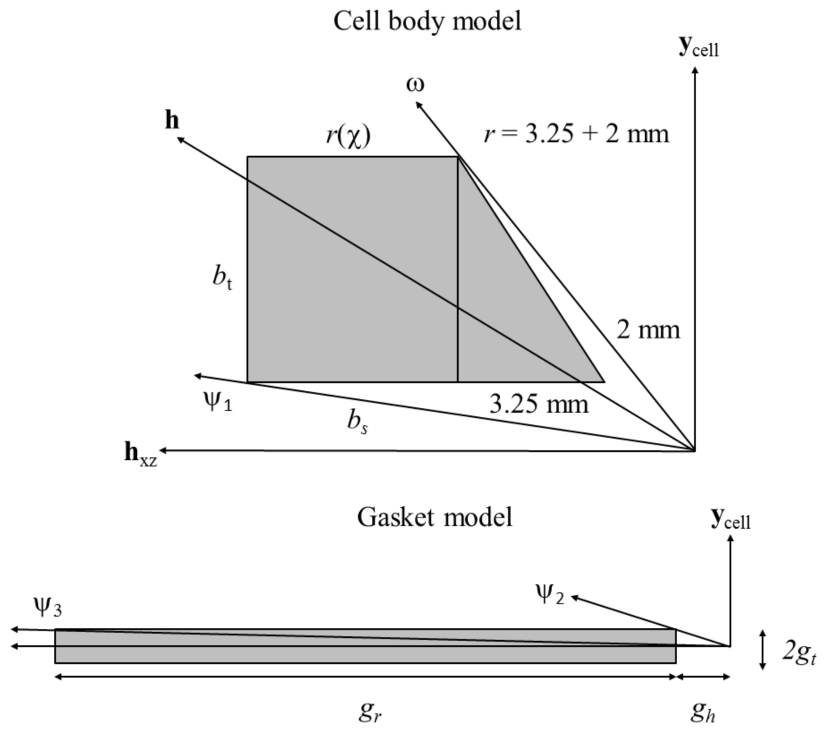

**ψ limits for gasket**	
$\psi_2 = \mathrm{atan}\left(\frac{g_h}{g_t}\right)$	Low-angle limit of gasket
$\psi_3 = \mathrm{atan}\left(\frac{g_r + g_h}{g_t}\right)$	High-angle limit of gasket

**ψ range**	**Expression for l_i**
$\psi \leq \omega$	$l_{i,d} = \frac{d_h}{\cos\psi}$

$\omega < \psi \leq \psi_1$	Diffracted ray has equation $$y_{\mathbf{h}} = \frac{x}{\tan(\psi)}$$ Intercepts are determine between $y_{\mathbf{h}}$ and the two possible entry points: Conic opening face: $$y_{cone} = \left(\frac{b_t}{r_{cone}}\right)x + b_s - \left(\frac{b_r d_r}{r_{cone}}\right)$$ Cell rear face: $$y_r = b_s$$ The point furthest from the origin is the first intercept. The second set of intercepts is determined between: Cell front face: $$y_f = b_s + b_t$$ Cell side wall: $$x = r + r(\chi)$$ The point closest to the origin is the second intercept. The path length $l_{i,cb}$ is the distance between these points.
$\psi_1 < \psi \leq 90°$	Ray passes between cell bodies, gasket attenuation only.

**Gasket terms**	**Expression for l_i**
$\psi_2 < \psi \leq \psi_3$	$l_{i,g} = \frac{g_t - (g_h \tan(90° - \psi))}{\sin(90° - \psi)}$
$\psi_3 < \psi \leq 90°$	$l_{i,g} = \frac{g_r}{\sin(\psi)}$

**Linear attenuation functions $\mu(\lambda)$**	
Diamond	$\mu_d(\lambda) = 0.0005\lambda$
BERYLCO-25 ($Cu_{0.87}Be_{0.13}$, density 8.249 g cm^{-3})	$\mu_{cb}(\lambda) = 0.1611\lambda + 0.042$
Iron	$\mu_g(\lambda) = 0.1208\lambda + 0.0332$
